# Supplementary material for: Intranasal Delivery of Quillaja brasiliensis Saponin-Based Nanoadjuvants Improve Humoral Immune Response of Influenza Vaccine in Aged Mice
Source: Vaccines (Basel). 2024 Aug 9;12(8):902. doi: 10.3390/vaccines12080902 (PMC11360193; doi:10.3390/vaccines12080902)
Supplement: Supplementary file 1 [file vaccines-12-00902-s001.zip › vaccines-3103159-supplementary.pdf]

Supporting Information

Intranasal delivery of *Quillaja brasiliensis* saponin-based nanoadjuvants improve humoral immune response of influenza vaccine in aged mice.

Fernando Silveira[1,*], Florencia García[1], Gabriel García[1], Alejandro Chabalgoity[1], Silvina Rossi[2] and Mariana Baz[3].

[1]Departamento de Desarrollo Biotecnológico, Instituto de Higiene, Facultad de Medicina, Universidad de la República. Av. Alfredo Navarro 3051, Montevideo, Uruguay.

[2]Departamento de Bioquímica Clínica, Instituto Polo Tecnológico, Facultad de Química, UdelaR, Ramal "José D'Elía" Ruta 101 y 8, CP. 91000 Canelones, Uruguay

[3]Department of Microbiology, Infectious Disease and Immunology, Faculty of Medicine, Université Laval, Quebec City, Québec, Canada.

***Corresponding author:** fsilveira@higiene.edu.uy

Materials and Methods

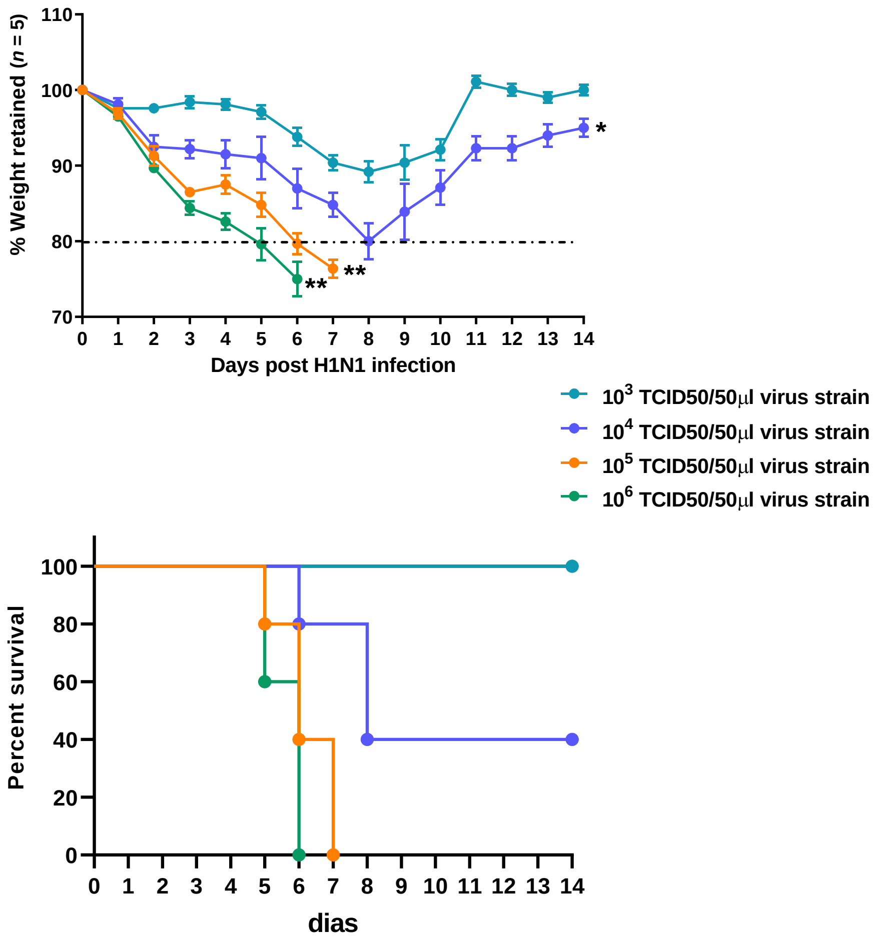

Supplementary figure S1. **Challenge test in adult female BALB/c mice.** 8-week-adult mice were intranasally challenged with different dose of A/Uruguay/897/2018 (H1N1)pdm09-like virus. All animals in each group (n = 5) were intranasally challenged with 1 × 10^6, 1 × 10^5, 1 × 10^4 and 1 × 10^3 tissue culture infectious dose (TCID)50/50μL of A/Uruguay/897/2018 (H1N1)pdm09-like virus. The animals per group were monitored for weight loss (A) (represented by mean and error) and mortality (B). Statistical analyses were performed using the nonparametric Kruskal–Wallis test with uncorrected Dunn's post-test for multiple comparisons. Probability of survival as determined by Log-rank (Mantel-Cox) test compared between groups. Statistical analyses were performed using the nonparametric Kruskal–Wallis test with uncorrected Dunn's post-test for multiple

comparisons. Percentage survival was determined by a log-rank (Mantel–Cox) test compared to 10^3 TCID50µL/50µL virus strain. Significant differences are indicated: *($P < 0.05$), **($P < 0.01$) and ***($P < 0.001$)
